# Resistance to ceftazidime-avibactam in a KPC-2–producing *Klebsiella pneumoniae* caused by the extended-spectrum beta-lactamase VEB-25

**DOI:** 10.1007/s10096-023-04582-0

**Published:** 2023-03-06

**Authors:** Jacqueline Findlay, Laurent Poirel, Maxime Bouvier, Valeria Gaia, Patrice Nordmann

**Affiliations:** 1 Medical and Molecular Microbiology, Faculty of Science and Medicine, University of Fribourg, Fribourg, Switzerland; 2 Swiss National Reference Center for Emerging Antibiotic Resistance (NARA), University of Fribourg, Fribourg, Switzerland; 3 Institute for Microbiology, University of Lausanne and University Hospital Centre, Lausanne, Switzerland; 4 Servizio Di Microbiologia EOLAB, Ente Ospedaliero Cantonale, Bellinzona, Switzerland

**Keywords:** *Klebsiella pneumoniae*, Ceftazidime-avibactam, KPC, VEB

## Abstract

Carbapenem-resistant Enterobacterales, including KPC-producing *Klebsiella pneumoniae*, represent a major threat to public health due to their rapid spread. The beta-lactam/beta-lactamase inhibitor (BL/BLI) combination ceftazidime-avibactam (CAZ-AVI) has recently been introduced and shown to exhibit excellent activity toward multidrug-resistant KPC-producing Enterobacterales strains. However, CAZ-AVI-resistant *K. pneumoniae* isolates are being increasingly reported, mostly corresponding to producers of KPC variants that confer resistance to CAZ-AVI but at a cost of carbapenem resistance. We have characterized here, both phenotypically and genotypically, a clinical CAZ-AVI- and carbapenem-resistant KPC-2 K*. pneumoniae* isolate co-producing the inhibitor-resistant extended-spectrum beta-lactamase VEB-25.

## Introduction

VEB-1 (Vietnamese extended-spectrum β-lactamase) was initially described in 1999 in an *Escherichia coli* isolate obtained from a Vietnamese patient hospitalised in Paris [1]. The enzyme was found to confer resistance to broad-spectrum cephalosporins and be plasmid-encoded and located within a class 1 integron [1]. Since then, this Ambler class A enzyme along with its variants have been predominantly described in Enterobacterales, *Pseudomonas* spp. and *Acinetobacter* spp., and have been reported globally [2, 3]. To date, 31 VEB variants have been identified (http://bldb.eu/).

Avibactam, a non-β-lactam-based diazabicyclooctane (DBO) molecule, used in combination with the third-generation cephalosporin ceftazidime, has been shown to have potent inhibitory activity against Ambler classes A and C and to some class D β-lactamases [4]. Hence, the ceftazidime-avibactam (CAZ-AVI) combo is being used as last-resort option for the treatment of serious infections caused by carbapenem-resistant Enterobacterales, especially those producing KPC-type carbapenemases against which it usually exhibits excellent activity [5]. However, in recent years, resistance to CAZ-AVI has emerged among KPC-producing *Klebsiella pneumoniae* (KPC-Kp), usually mediated via the production of KPC variants and at the cost of carbapenem resistance that is subsequently almost fully lost [6]. VEB variants are known to be effectively inhibited by avibactam [7]. However, a novel VEB variant, namely VEB-25, which was initially reported in 2019 from two KPC-2-producing *K. pneumoniae* isolates obtained from patients hospitalized in two Greek hospitals, has been reported to be a source of CAZ-AVI resistance [8]. This study identified these two isolates as belonging to Sequence Types ST147 and ST258, harbouring the *bla*_VEB-25_ gene on ca. 150 kb InC plasmid and a ca. 170 kb IncC/IncR plasmid, respectively. A subsequent study reported an outbreak within two ICUs in a single hospital in Athens, which affected seven patients within a 5-week period in 2019, and the strain responsible was identified as an ST147 *K. pneumoniae* isolate co-producing KPC-2 [9]. To our knowledge, this variant has only been reported in Greece so far.

We report here the phenotypic and genotypic characterisation of a CAZ-AVI-resistant *K. pneumoniae* isolate co-producing KPC-2 and VEB-25, recovered from a patient in Switzerland.

## Materials and methods

### Bacterial isolates

A carbapenem-resistant *K. pneumoniae* isolate, N3418, was sent to the Swiss National Reference Centre for Emerging Antibiotic Resistance (NARA) for further investigation. Species identification was confirmed using the EnteroPluri-Test (Liofilchem https://www.liofilchem.com) and UriSelect 4 agar (Bio-Rad, Cressier, Switerland). The isolate was subjected to the detection of carbapenemase activity by the Rapidec Carba NP test (bioMérieux) and then to NG-Test CARBA 5 test (NG Biotech), according to the manufacturer’s instructions. KPC alleles were identified by PCR amplification and Sanger sequencing.

### Antimicrobial susceptibility testing

MICs of antimicrobial agents were determined by broth microdilution in cation-adjusted Mueller–Hinton broth (BioRad) for all antibiotics. The preparation of the different BL/BLIs, namely, CAZ-AVI, CAZ-relebactam (CAZ-REL), CAZ-tazobactam (CAZ-TAZ), CAZ-clavulanic acid (CAZ-CLA), CAZ-vaborbactam (CAZ-VAB), aztreonam-AVI (AZT-AVI), imipenem-REL (IPM-REL), meropenem-VAB (MEM-VAB), piperacillin-TAZ (PIP-TAZ), were performed according to the CLSI guidelines [10], with a fixed concentration of the inhibitors at 4 mg/L for AVI, REL, TAZ and CLA, and 8 mg/L for VAB.

### Sequence analyses

The *bla*_VEB-1_ and *bla*_VEB-25_ alleles were amplified using primers VEB-Fw (5′-GATGATGAGCTCGCAGTCGCCCTAAAACAAAG-3) and VEB-Rev (5′-GATGATGGATCCCAAATTGCACTTCAACCCGC-3′). Sequencing of the amplicons and recombinant plasmids was performed by Microsynth AG (Balgach, Switzerland).

### Cloning experiments

The *bla*_VEB_ alleles were amplified using the above primers, and cloned into pCR-Blunt II-TOPO (Invitrogen, ThermoFisher), before transformation into *Escherichia coli* Top10. Transformants were selected on plates supplemented with kanamycin (50 mg/L). Successful transformants were confirmed by amplification and sequencing of the *bla*_VEB_ alleles.

### Conjugation experiments and plasmid transformation

Mating-out assays of the *bla*_VEB-25_-harbouring plasmid was attempted using a sodium azide-resistant *E. coli* J53 as recipient, as previously described [11], with selection on sodium azide at 100 mg/L and ceftazidime-avibactam at 8/4 mg/L. Following failure of conjugation attempts, plasmid pVEB-25_IncC was transformed into *E. coli* Top10 by electroporation and selected on LB agar plates containing ceftazidime-avibactam at 8/4 mg/L. Transformants were confirmed by the amplification and subsequent sequencing of the *bla*_VEB-25_ and IncC *repA* gene.

### Whole genome sequencing (WGS)

WGS was performed using both short and long-read sequencing technologies as previously described, using either a MiSeq (Illumina) or MinION Mk1C (Oxford Nanopore Technologies, Oxford, UK) platforms [11].

Assemblies were performed using the Shovill pipeline (https://github.com/tseemann/shovill) and contigs were annotated using Prokka [12]. STs, the presence of resistance genes and plasmid replicon types were determined using MLST 2.0, ResFinder 4.1 [13] and PlasmidFinder 2.1 [14], on the Center for Genomic Epidemiology platform (https://cge.cbs.dtu.dk/services/). Long-read sequencing reads were trimmed and corrected using Canu [15]. Hybrid assembles, using both short and long-read data, were performed using UniCycler [16].

### *IC*_*50*_* measurements*

IC50 measurements were performed as previously described [17]. Briefly, β-lactamases from crude extracts were prepared and used for the measurement of the specific activity in 100 mM sodium phosphate (pH 7.0). Measurements were performed in a Genesys 10S UV/VIS spectrophotometer (Thermo Scientific) spectrophotometer using a wavelength of 262 nm for cephalothin. The 50% inhibitory concentration (IC_50_) for KPC variants was determined as the concentration of clavulanic acid (CLA), tazobactam (TAZ), REL, AVI, or VAB, that reduced the hydrolysis rate of 100 μM cephalothin (CEF) by 50%. Extracts were preincubated with inhibitor for 3 min prior to the addition of CEF. All measurements were performed in triplicate.

## Results and discussion

### Phenotypic and genotypic profiling of N3418

*K. pneumoniae* isolate N3418 was obtained from the urine of an 84-year-old male who had previously been hospitalised in Greece but had not previously received CAZ-AVI treatment. Susceptibility testing showed that N3418 was resistant to all beta-lactams tested, including the carbapenems and cefiderocol (FDC), in addition to the BL/BLI combination CAZ-AVI. The isolate remained susceptible to IPM-REL and MEM-VAB, but exhibited resistance to colistin (COL; MIC of 16 mg/L) (Table 1). N3418 was also resistant to ATM-AVI (using a provisional breakpoint of 4 mg/L), and high MICs to unconventional BL/BLI combinations CAZ-REL (128 mg/L, CAZ-CLA (256 mg/L), and CAZ-VAB (32 mg/L). PCR and Sanger sequencing identified the KPC-2 variant that could therefore not explain the resistance to CAZ-AVI. To ascertain the mechanism of CAZ-AVI resistance, the isolate was subjected to WGS which subsequently led to the identification of *bla*_VEB-25_. WGS identified that this isolate belonged to ST323 and harboured the *bla*_KPC-2_, *bla*_VEB-25_, and *bla*_OXA-10_ beta-lactamase genes (Table 2). ST323 has been identified as one of the most dominant lineages associated with ESBL phenotype in Australia, but is not related to the ST147 and ST258 previously identified as VEB-25 producers in Greece [17]. Plasmid replicon type analyses revealed a number of replicon types present, comprising Col440II, ColRNAI, IncC, IncFIB(pKPHS1), IncFIB(pQIL), IncFII(K), IncFII(pKP91), and IncR. Using a combination of long and short read sequencing, we could identify that the *bla*_KPC-2_ gene was encoded on a ~ 100 kb FIB(pQIL) plasmid and *bla*_VEB-25_ was encoded on a ~ 114 kb IncC plasmid. A deletion of the *mgrB* gene (involved in lipopolysaccharide synthesis) and disruption into the *fiu* gene were also found, likely contributing to COL and FDC resistance, respectively [19, 20].

**Table 1:** Susceptibility testing resulting of N3418 and recombinant *E. coli* strains producing VEB-1 and VEB-25

MICs (mg/L)																			
Strain	AMX	AMC	PIP	PTZ	ETP	IPM	MEM	FOX	FEP	CAZ	CAZ-AVI	CAZ-REL	CAZ-TAZ	CAZ-CLA	CAZ-VAB	ATM	ATM-AVI	FDC	COL
N3418	> 256	> 256	> 256	> 256	128	32	64	128	64	> 256	128	128	256	256	32	256	64	64	16
*E. coli* Top10	4	4	2	2	≤ 0.03	0.125	≤ 0.03	4	≤ 0.03	0.25	0.25	0.25	0.25	0.25	0.25	0.125	0.125	0.125	ND
*E. coli* Top10/pTOPO-VEB-1	> 256	4	128	2	≤ 0.03	0.125	≤ 0.03	4	16	> 256	4	4	4	1	32	256	0.5	2	ND
*E. coli* Top10/pTOPO-VEB-25	> 256	8	128	4	≤ 0.03	0.125	≤ 0.03	4	16	> 256	128	128	32	4	32	256	64	2	ND
*E. coli* Top10 pVEB-25_IncC Tf	> 256	16	16	2	≤ 0.03	0.125	≤ 0.03	4	1	256	64	64	8	2	8	32	16	2	ND

*AMX*, amoxicillin; *AMC*, amoxicillin-clavulanic acid; *PIP*, piperacillin; *PTZ*, piperacillin-tazobactam; *ETP*, ertapenem; *IPM*, imipenem; *MEM*, meropenem; *FOX*, cefoxitin; *FEP*, cefepime; *CAZ*, ceftazidime; *AVI*, avibactam; *REL*, relebactam; *CLA*, clavulanic acid; *TAZ*, tazobactam; *VAB*, vaborbactam; *ATM*, aztreonam; *FDC*, cefiderocol; *COL*, colistin

**Table 2:** Genotypic characteristics of N3418 and pVEB-25_IncC

Strain/plasmid	ST	Resistance genes	Replicons types	Size (bp)
N3418	323	*bla*_KPC-2_, *bla*_VEB-25_, *bla*_OXA-10_, *bla*_SHV-1_, *aph(3’)-la*, *aph(3″)-lb*, *aph(6)-ld*, *aadA1*, *aadB*, *arr-2*, *qnrS1*, *oqxA*, *oqxB*, *sul2* (× 2), *tetA*, *∆qacA*, *∆sul1*, *cmlA5*, *fosA5*, *∆fosA7*, *dfrA23*	Col440II, ColRNAI, C, FIB(pKPHS1), FIB(pQIL), FII(K), FII(pKP91), R	NA
pVEB-25_IncC	NA	*bla*_VEB-25_, *bla*_OXA-10_, *tetA*, *∆qacE*, *cmlA5*, *dfrA23*, *∆sul1*, *sul2* (× 2), *qnrS1*, *arr-2*, *aph(3’)-la*, *aph(3″)-lb* (× 2), *aph(6)-ld*, *aadA1*, *aadB*	C	114,198

*NA*, not applicable

### Phenotypes of the recombinant E. coli strains producing VEB-25 and VEB-1, and relative enzyme kinetic measurements

Susceptibility testing of recombinant *E. coli* strains producing VEB-1 and VEB-25 showed that, relative to VEB-1, the VEB-25 recombinant strain exhibited increased MICs for AMC, PTZ, CAZ-AVI, CAZ-REL, CAZ-TAZ, CAZ-CLA, and ATM-AVI (Table 1). Fold changes for each BLBI ranged from 2- to 128-fold, with the greatest change being observed between the BL/BLI combination encompassing the beta-lactams (CAZ and ATM) and the DBOs, AVI and REL.

IC_50_ assays showed that the inhibitory activity of AVI, REL, clavulanic acid and tazobactam were considerably decreased against VEB-25 compared to VEB-1, correlating with the susceptibility testing results (Table 3). The Lys234 residue is conserved in most class A beta-lactamases [21], and has been indicated as critical for the inhibitory activity of avibactam toward KPC enzymes. Accordingly, this finding correlates with what has been observed from in vitro mutation studies in KPC enzymes [22]. Noteworthy, in silico Genbank analysis identified the VEB-20 variant exhibiting the same Lys234Arg substitution as VEB-25, encoded on the chromosome of a *Pseudomonas aeruginosa* recovered from France (GenBank accession number: NG_063894). However, the phenotype conferred by this enzyme remains unknown.

**Table 3:** Inhibitory concentrations of beta-lactamase inhibitors against VEB-1 and VEB-25

IC50 (µM)					
Enzyme	AVI	REL	CLA	TAZ	VAB
VEB-1	0.9	2.4	0.05	0.05	1.1
VEB-25	346.6	340.3	0.5	1.1	1.0

*AVI*, avibactam; *REL*, relebactam; *CLA*, clavulanic acid; *TAZ*, tazobactam; *VAB*, vaborbactam

### Plasmid pVEB-25_IncC encoding VEB-25

**Fig. 1:**
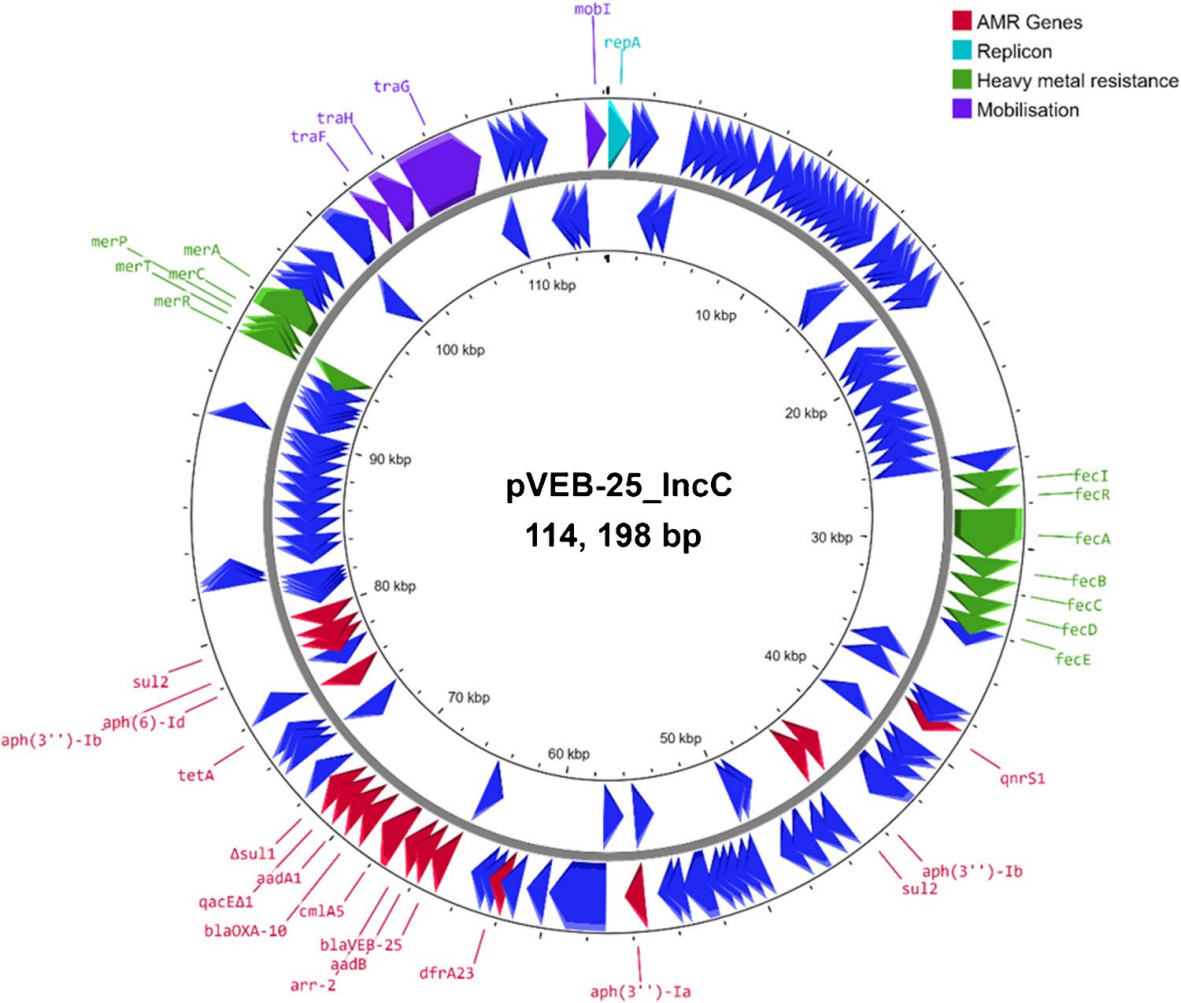
Plasmid pVEB-25_IncC

The natural *bla*_VEB-25_-harbouring plasmid pVEB-25_IncC was transformed into *E. coli* Top10. Susceptibility testing showed that pVEB-25_IncC conferred resistance to a range of beta-lactams including CAZ, ATM, AMX, PIP and the BL/BLIs CAZ-AVI, PTY and AMC (Table 1). Plasmid pVEB-25_IncC was found to be an IncC replicon type and 114,198 bp. In addition to *bla*_VEB-25_, the plasmid also encoded 15 further resistance genes encoding resistance to multiple antibiotic classes; *bla*_OXA-10_ (beta-lactams), *tetA* (tetracycline efflux pump), *qnrS1* (quinolones), *cmlA5* (phenicols), *arr-2* (rifiampicin), *dfrA23* (trimethoprim), *sul1* and 2 copies of *sul2* (sulphonamides), and six aminoglycoside resistance genes (*aph(3″*)-*Ia*, *ant*(*2″*)-*Ia*, *ant*(*3″*)-*Ia*, *aph*(*6*)-*Id* and 2 copies of *aph*(*3″*)-*Ib*) (Fig. 1). The ~ 9 kb multidrug resistance region harbouring *bla*_VEB-25_ comprised; IS*10A*, *bla*_VEB-25_, *aadB*, *arr-2*, *cmlA5*, *bla*_OXA-10_, *aadA1*, *qacE∆1*, *∆sul1*, IS*26*. This is partially similar to the ~ 14 kb multidrug resistance region previously described by Voulgari et al., however lacking a ~ 5 kb region that includes *rmtB1* and *bla*_TEM-1_ [8]. However, pVEB-25_IncC also harboured a mercury resistance operon (*merACPTR*) and citrate-dependent iron (III) uptake system (*fecIRABCDE*), in addition to a the HigAB type II toxin-antitoxin system that is typical of IncC plasmids [23]. Interestingly, the *fecIRABCDE* gene cluster has recently been linked to reduced susceptibility to FDC and its presence, alongside the *fiu* gene disruption, may explain the FDC resistance levels observed here [24]. Conjugation attempts were unsuccessful; however, pVEB-25_IncC could be transferred *to E. coli* Top10 by electroporation. Analysis of the plasmid sequence using Mob-Typer [25] predicted that the plasmid was mobilizable but not conjugative, and further analysis of the plasmid content revealed that it contained only a partial transfer (*tra*) region, comprising *traF*, *traG* and *traH*.

## Conclusions

We characterized here a VEB variant responsible for CAZ-AVI resistance that has previously only been rarely reported in Greece. The amino acid change, Lys234Arg, that differentiates VEB-1 from VEB-25, clearly plays an important role in the activity of beta-lactamase inhibitors — most notably the DBO compounds. Overall, VEB-mediated resistance to CZ-AVI in *K. pneumoniae* has been reported rarely but should be considered when encountering resistant isolates.

## Data Availability

Sequence data from this study was submitted to the National Center for Biotechnology Information’s Sequence Read Archive (BioProject no. PRJNA922098). The plasmid sequence of pVEB-25_IncC has been submitted to GenBank with accession number OQ362291.
